# Repeated Resistance Training Reveals the Reproducibility of Muscle Strength and Size Responses Within Individuals

**DOI:** 10.1002/ejsc.70095

**Published:** 2025-11-27

**Authors:** Aapo Räntilä, Eeli J. Halonen, Esko J. Tiainen, Sasu O. Kaasinen, Juha J. Hulmi, Juha P. Ahtiainen

**Affiliations:** ^1^ Faculty of Sport and Health Sciences University of Jyväskylä Jyväskylä Finland

**Keywords:** detraining, heterogeneity, minimal detectable change, muscle hypertrophy, one repetition maximum, ultrasound, vastus lateralis

## Abstract

This study investigated whether the responses of upper and lower limb muscle strength and size to resistance training (RT) are reproducible across two RT periods. Untrained males and females (age 32 ± 5 years) were randomly assigned to RT (*n* = 20) or a control group (*n* = 27). The RT group completed two identical 10‐week RT periods separated by 10 weeks of detraining. The control group underwent a 10‐week non‐RT period. Before and after each period, the muscle cross‐sectional area (CSA) of the vastus lateralis (VL) and biceps brachii (BB) was measured by ultrasound. One‐repetition maximum (1RM) was measured in leg press (LP) and biceps curl (BC). Minimal detectable changes were determined. The RT group showed greater gains (*p* < 0.001) in muscle size and strength than the control group, with variability observed among individual responses. The reproducibility of responses within‐participants was demonstrated by correlations (*p* ≤ 0.001) between the first and second RT periods in VLCSA (*r* = 0.697), BBCSA (*r* = 0.761), and LP1RM (*r* = 0.671), with a trend for BC1RM (*r* = 0.393 and *p* = 0.095). Nonresponders were identified, but none were detected in both RT periods for more than one variable. First RT correlated negatively (*p* < 0.05) with subsequent detraining in BBCSA (*r* = −0.673) and LP1RM (*r* = −0.488), with a trend for VLCSA (*r* = −0.422 and *p* = 0.072). In conclusion, responses to RT are reproducible when RT is repeated, indicating that the individuality of the training response has a physiological origin. Nonresponsiveness is rare and should not be justified solely by one variable. Responses to detraining suggest that greater RT gains may also diminish faster.

## Highlights

- Resistance training induced individual variability in muscle size and strength responses. Standard deviation of individual response values exceeded zero for all variables, confirming heterogeneity beyond measurement error and random noise. Findings demonstrate that responses to training and detraining reflect consistent biological adaptations.
- Correlation analyses showed that responsiveness to resistance training is consistent when the protocol is repeated after detraining.
- Extreme responders in muscle size and strength were consistently observed, whereas nonresponders were rare and not replicated across variables.
- Participants with greater initial gains in muscle size and strength tended to lose more during detraining. This suggests that individuals who gain muscle mass to a greater extent may also lose it rapidly when training ceases.

## 1 Introduction

Individual responses to a given resistance training (RT) intervention have received increasing scientific interest. The reported range of individual responses to RT interventions has proven to be enormous, from −11%–30% (Ahtiainen et al. 2016), −2–59% (Hubal et al. 2005), and −3–18% (Erskine et al. 2010) for muscle size and, from −8–60% (Ahtiainen et al. 2016) and 0%–250% (Hubal et al. 2005) for muscle strength. Intraindividual variation can account for a significant portion of the observed differences in training responses (Williamson et al. 2017, 2018). It refers to the differences in measurements obtained from the same participant under standardized conditions (Chrzanowski‐Smith et al. 2020), often termed day‐to‐day or within‐participant variation, indicating the reproducibility of an observation. In the context of RT responses, it is more relevant to consider intraindividual variability in pre‐to‐post differences when the same intervention is repeated (Chrzanowski‐Smith et al. 2020).

Measured exercise training responses are influenced by variability arising from three primary sources: measurement error, biological error, and biological variation (Atkinson and Nevill 1998; Hecksteden et al. 2015; Senn et al. 2011). To distinguish intervention‐induced changes from intraindividual variations caused by these factors (Solomon 2018; Voisin et al. 2019), the most robust approach is to perform replicated interventions with the same participants (Senn et al. 2011). Although several studies have conducted repeated RT interventions to examine the muscle memory phenomenon (Cumming et al. 2024; Seaborne et al. 2018), these investigations have not explored inter or intraindividual variability in muscle size and strength responses nor have they examined individual responses to detraining. Our prior research has shown that individuals exhibiting larger gains in muscle size and strength during training experienced greater losses during detraining (Räntilä et al. 2021). Similarly, as individual responses to resistance training (Roberts et al. 2018), this may be explained by the molecular responses to detraining. However, studies have not focused on addressing the molecular mechanisms of high or low responders to detraining (Cumming et al. 2024; Hulmi et al. 2025; Seaborne et al. 2018). Notably, as examined in the subgroup of present participants, many of the proteins upregulated during the first RT period were also affected during the second RT period, suggesting consistent molecular responsiveness across repeated training cycles (Hulmi et al. 2025). Supporting this notion, endurance training research has also demonstrated good reproducibility of gene expression changes in response to repeated training bouts (Lindholm et al. 2016). Still, the question remains whether the changes in muscle size and strength that occur after one RT period will be repeated similarly during the next RT period within the individuals.

This study employed a training‐detraining‐retraining design to examine variation within participants across repeated RT periods in muscle strength and size (i.e., intraindividual variation). Additionally, nonresponsiveness to RT and detraining responses were investigated. To support these analyses, RT response heterogeneity was assessed by including a nontraining control group. The hypotheses of the study were that heterogeneity in RT responses, including nonresponders, can be observed and that individual responses in the first RT intervention period are reproducible in the second identical RT period. We also hypothesized that we would find individual differences in responsiveness during the detraining phase.

## 2 Methods

### 2.1 Participants

The present study is a part of a larger research project (Halonen et al. 2024). In this study, fifty‐five participants (both males and females) were recruited from the Central Finland region through advertisements on notice boards, websites, and emails. Eligible participants were aged 18–40 years, had no regular RT history (< 10 RT sessions per year), were not engaged in systematic endurance training in the past 6 months (> two endurance sessions per week, each lasting over 30 min), had a body mass (BM) index between 18.5 and 30 kg/m^2^, and were not currently using any anti‐inflammatory drugs. Exclusion criteria included medication affecting exercise responses or any condition limiting RT, such as respiratory, musculoskeletal, endocrinological, or psychological disorders. After a health assessment by a physician, each participant provided a written informed consent after being informed of potential risks, discomforts, and their right to withdraw at any time.

Of the 55 participants, 28 were randomized to the RT group and 27 to the control group. The study used a randomized, parallel‐group, and repeated‐measures design. Before baseline measurements, participants were randomly allocated in a 1:1 ratio to either the RT or control group by separately dividing male and female participants into the closest (matched) pairs according to a combined z‐score of BMI and age to ensure baseline homogeneity between groups. Eleven males and nine females completed the study (32.9 ± 5.9 years, 174.2 ± 9.7 cm, BM 78.2 ± 15.3 kg, and BMI of 25.7 ± 3.4 kg/m^2^) in the RT group, and 27 participants (14 males and 13 females) completed the control period (31.4 ± 4.3 years, 173.4 ± 10.5 cm, 74.0 ± 15.7 kg, and BMI of 24.8 ± 3.6 kg/m^2^), with no statistically significant differences between the groups. For the RT group, three dropouts during the first RT period were due to health/illness‐related reasons and one for unknown reasons. During the second RT period, four participants withdrew, three for health/illness‐related reasons and one due to time constraints. The sample size for detecting differences in muscle strength and size between the RT and control groups after the first 10‐week period was determined using the G*Power software (version 3.1.9.7). In our earlier 10‐week RT study with previously untrained participants of similar age as in this study, the pre‐ to postchanges in muscle size were 11.5 ± 13.3% and −2.7 ± 7.9% in the RT and control groups, respectively (Hulmi, Kovanen, et al. 2009). In terms of muscle strength, the changes were 13.4 ± 7.2% versus 2.9 ± 7.1% (Hulmi, Tannerstedt, et al. 2009). Based on effect sizes observed in that previous study and assuming 80% statistical power with a two‐tailed *α* level of 0.05, a sample size of 20 participants per group was deemed sufficient for between‐group comparisons in the present study, while also accounting for potential dropouts. The study received ethical approval from the University of Jyväskylä's ethics committee (857/13.00.04.00/2021) and was conducted in accordance with the Declaration of Helsinki. Personal data were managed according to the ethical and GDPR guidelines of the University of Jyväskylä. The study has been registered in ClinicalTrials.gov (NCT05553769).

### 2.2 Study Design

**FIGURE 1:**
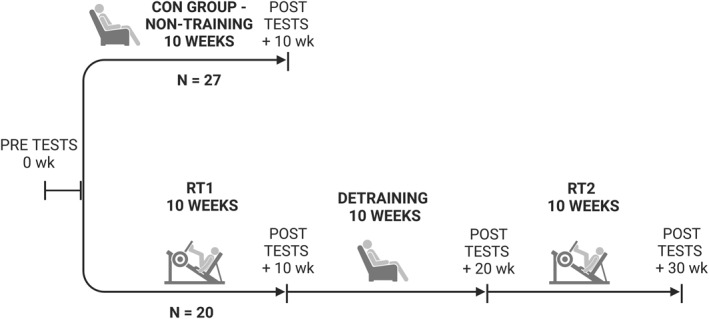
Representation of the study design. Numbers refer to weeks. CON = control and RT = resistance training.

After the participants were recruited, a pretest was administered. Subsequently, the control group commenced a 10‐week non‐RT phase, whereas the RT group began their first 10‐week RT period (RT1). In the control group, compliance with the nontraining instructions was verified using physical activity questionnaires. After 10 weeks, both groups participated in posttests. Following this, the RT group initiated a 10‐week detraining period, followed by a second 10‐week RT (RT2), which was identical to RT1. Muscle strength and size assessments were conducted after each 10‐week intervention period (Figure 1). The study could not be conducted as a blinded trial.

### 2.3 Resistance Training Program

During the RT, participants completed two similar RT sessions per week. The training protocol has been reported in detail previously (Halonen et al. 2024). In short, exercises were leg press (LP) (4 × 8–10 repetitions), knee extension (4 × 8–10 repetitions), Smith machine bench press (3 × 8–10 repetitions), barbell biceps curl (BC) (4 × 8–10 repetitions), and chest supported seated row (4 × 8–10 repetitions) with two‐minute rest periods between the sets. The participants performed 8–10 repetitions in each set with approximately 1–2 repetitions in reserve, except in the final set of each exercise in the second training session every week, when the last set was performed until volitional failure. For progression, the number of repetitions in the failure sets was used to adjust the training loads for the following week; if the number of performed repetitions exceeded 10, the load was increased, and if the repetitions were fewer than eight, the load was decreased. All training sessions were conducted at the laboratory of the University of Jyväskylä’s Faculty of Sport and Health Sciences and supervised to ensure correct exercise techniques.

### 2.4 Detraining

Participants were instructed to maintain their usual lifestyle during the 10‐week detraining period, refraining from RT, high‐intensity physical activity, and any other unaccustomed exercise. Compliance with these instructions was verified through physical activity surveys and email contact at mid‐period to confirm that participants adhered to the no‐RT guidelines.

### 2.5 Measurement of Muscle Cross‐Sectional Area

Muscle cross‐sectional area (CSA) of the vastus lateralis (VL) and biceps brachii (BB) was assessed using a B‐mode axial plane ultrasound (model SSD‐α10, Aloka, Tokyo, Japan) with a 10 MHz linear‐array probe (60 mm width) in extended‐field‐of‐view mode (23 Hz sampling frequency) (Ahtiainen et al. 2010). All imaging was performed by the same experienced researcher and conducted at least 48 h postexercise to avoid resistance exercise‐induced edema. Great care was used not to compress the probe while imaging. For VLCSA, participants lay supine with legs strapped to polystyrene supports to ensure stability. Anatomical landmarks were identified on the right leg, from the greater trochanter to the proximal edge of the patella, with 50% of this length marked and an axial line drawn on the skin. All distances were documented and photographed, and a custom‐made probe support maintained a perpendicular probe angle. One participant was excluded from the analysis for the VLCSA images due to insufficient image resolution.

BBCSA was measured with participants seated and their right arm supported at a 45° angle to the torso on polystyrene supports. The distance from the acromion process to the elbow joint's central point was measured, and a point at one‐third this distance from the elbow was marked on the skin. An axial line was drawn, and a loose strap was secured around the arm above the line to guide the probe. Image analysis was conducted manually using the ImageJ software (version 1.54). For VLCSA, three images were analyzed and the mean of the two closest values was used. For BBCSA, a novel method in our laboratory, 3 to 6 images of the biceps brachii were analyzed at each time point by manually tracing the visible muscle borders (excluding the brachialis). If the coefficient of variation (CV%) was less than 5%, the average of all images was used. If the CV% exceeded 5%, the most divergent image was excluded to achieve a CV% below 5% and the average of the remaining images was used. The test–retest CV% for VLCSA and BBCSA were 1.6% and 2.3%, respectively (Halonen et al. 2024).

### 2.6 Muscle Strength Tests

Dynamic maximum strength was assessed using one‐repetition maximum (1RM) tests for the leg press and barbell biceps curl. Before testing, participants completed a standardized warm‐up, followed by progressively increasing loads to determine their 1RM. Rest intervals of 3 min were maintained between attempts, and typically, 3–5 attempts were required. The leg press 1RM was performed on a horizontal leg press machine (David, F210, Finland). The load was first assisted to the starting position, 180° knee angle, allowing the eccentric phase to begin with straight legs. Participants paused at approximately 65° knee angle at the bottom position and initiated the concentric phase on the researcher's command. The knee angle in the bottom position was measured at the beginning of the intervention using a manual goniometer, and the seat and footplate settings were then adjusted accordingly. The barbell biceps curl 1RM was conducted using custom‐made equipment with adjustable padding to support the lumbar spine and upper back. Participants were secured with a leather belt, and the movement was performed in a concentric motion, maintaining contact with the padding for the repetition to be accepted. Each repetition had to start with straight arms and finish with complete elbow flexion. The test–retest CV% for leg press and biceps curl 1RM were 3.1% and 4.2%, respectively (Halonen et al. 2024).

During the first training session of both 10‐week RT periods, 3–5 repetition maximum (RM) tests were conducted for knee extension, Smith machine bench press, and chest‐supported seated row exercises to determine starting RT loads (Halonen et al. 2024).

### 2.7 Body Mass Measurement

Body mass was assessed using the InBody770 bioelectrical impedance analyzer (Biospace Co. Ltd., Seoul, Korea) with a multifrequency current. Measurements were conducted according to the manufacturer's instructions in the morning after overnight fasting. Given the variations in participants' anthropometric characteristics, strength test results were normalized to body mass for the leg press and biceps curl to account for these differences. Since the primary aim was to equalize strength test results between males and females of different body sizes, without allowing variations in body mass during the intervention to affect the interpretation of the results, the baseline body mass was used consistently throughout the study for normalization purposes.

### 2.8 Identifying Individual Differences

First, we compared the variance of the pre–post differences during RT1 between the RT group (SD_EX_) and the control group (SD_CON_). If the variance (SD^2^) in the RT group exceeded that in the control group, it indicated evidence of individual response variation on average (W. G. Hopkins et al. 1999; Senn et al. 2011). SD of individual response (SD_IR_) is calculated using the following equation (Atkinson and Batterham 2015; G. Hopkins 2015; Williamson et al. 2017):

$${\text{SD}}_{\text{IR}}=\sqrt{{\left({\text{SD}}_{\text{EX}}\right)}^{2}-{\left({\text{SD}}_{\text{CON}}\right)}^{2}}$$

where SD_EX_ and SD_CON_ are the standard deviations of change scores in the RT and control groups, respectively. Positive SD_IR_ values indicate the presence of interindividual differences in trainability. In contrast, SD_IR_ values of zero or less suggest that variability in observed responses may be attributable to measurement error or biological variability rather than true individual differences. Confidence limits for SD_IR_^2^ and its standard errors (SEs) were determined using the approach outlined in (G. Hopkins 2015). Individual‐level responses were determined when the SD_IR_ indicated individual differences at the group level. To determine the smallest changes that represent a meaningful response to RT, the minimal detectable change (MDC) was calculated for each variable, following the principles proposed by (Swinton et al. 2018). The participant was classified as a nonresponder if the change during the RT period was lower than MDC, a responder when higher than MDC, and an extreme responder if the change exceeded three times the MDC in both RT periods. MDC incorporates the typical error of change scores (TEΔ), which were calculated as the standard deviation of the difference scores (SDd) derived from the control group's results between weeks 0 and 10 (Charter and Feldt 2001; W. G. Hopkins 2000; Weir 2005):

$$\text{TE}\Delta =\text{SDd}/\sqrt{2}$$

Then, MDC was calculated with the following formula (Beaton 2000):

$$MDC=TE\Delta \;x\;1.96\;x\;\sqrt{2}$$

### 2.9 Statistical Analysis

Statistical analyses were performed using SPSS Statistics version 24 (IBM Corp., New York, NY, USA). Figures were made with Microsoft Excel (version 2410) and https://BioRender.com. The differences in all dependent variables between the RT and control groups in responses from 0 to 10 weeks and changes over a training‐detraining‐retraining intervention, and the differences in responses between the intervention periods in the RT group were analyzed using the linear mixed model (dependent variable: measured value; fixed effect: group or measurement time point or variable; and random effect: subject ID). Pearson's product‐moment correlation coefficients (*r*) were used to explore relationships between variables. A *p*‐value of less than 0.05 was considered statistically significant.

## 3 Results

### 3.1 Identifying Individual Differences in Repeated Resistance Training

The interaction between group (RT vs. control) and time point (0‐week pretests vs. 10‐week posttests) was significant for all variables, indicating that the changes from pre‐to post‐10‐week tests differed between the RT and control groups. Specifically, for VLCSA (16.0 ± 8.4% and 1.5 ± 4.1% in the RT and control groups, respectively), the interaction was significant, F(1, 44) = 49.06 and *p* < 0.001; for BBCSA (17.5 ± 6.5% vs. 3.1 ± 2.8%), F(1, 45) = 73.82 and *p* < 0.001; for LP1RM/BM (21.7 ± 12.6% vs. 2.9 ± 4.5%), F(1, 45) = 62.42 and *p* < 0.001; and for BC1RM/BM (25.4 ± 19.0% vs. 4.5 ± 6.2%), F(1, 45) = 52.84 and *p* < 0.001 (Table 1). Statistically significant increases (*p* < 0.001) were observed only in the RT group in muscle size and strength after both RT periods. Responses were greater in RT1 compared to RT2 in LP1RM/BM (F(1, 19) = 22.1 and *p* < 0.001) and BC1RM/BM (F(1, 19) = 14 and *p* = 0.001), whereas no differences were observed in VLCSA and BBCSA. Moreover, differences were observed between the RT and control groups (*p* < 0.001) after the first 10‐week period across all variables (Table 1).

**TABLE 1:** Muscle size and strength in experimental (*n* = 20) and control (*n* = 27) groups.

		PRE (week 0)	After RT1 (week 10)	After DT (week 20)	After RT2 (week 30)
VLCSA (cm^2^)	Exp _(n = 19)_	26.2 ± 5.9	30.3 ± 7.2^a^	27.2 ± 6.0^a^	31.3 ± 7.3^a^
	Con	26.5 ± 5.8	26.9 ± 5.8^b^		
LP1RM/BM (kg/kg)	Exp	2.12 ± 0.48	2.54 ± 0.49^a^	2.41 ± 0.51^c^	2.70 ± 0.53^a^
	Con	2.19 ± 0.35	2.24 ± 0.32^b^		
BBCSA (cm^2^)	Exp	9.1 ± 3.1	10.6 ± 3.5^a^	9.8 ± 3.3^a^	11.3 ± 3.6^a^
	Con	8.8 ± 3.2	9.1 ± 3.3^b^		
BC1RM/BM (kg/kg)	Exp	0.36 ± 0.09	0.44 ± 0.09^a^	0.43 ± 0.10	0.47 ± 0.10^a^
	Con	0.37 ± 0.08	0.39 ± 0.08^b^		

Abbreviations: 1RM = one repetition maximum, BB = biceps brachii, BC = biceps curl, BM = body mass, Con = nontraining control group, CSA = cross‐sectional area, DT = detraining, Exp = experimental resistance training group, LP = leg press, RT1 = first resistance training period, RT2 = second resistance training period, and VL = vastus lateralis.

^a^ Denote statistically significant (*p* < 0.001) changes compared to the previous time point.

^b^ Indicate statistically significant differences (*p* < 0.001) compared to the experimental group for absolute changes during the first 10‐week intervention period.

^c^ Denotes statistically significant (*p* < 0.05) changes compared with the previous time point.

**FIGURE 2:**
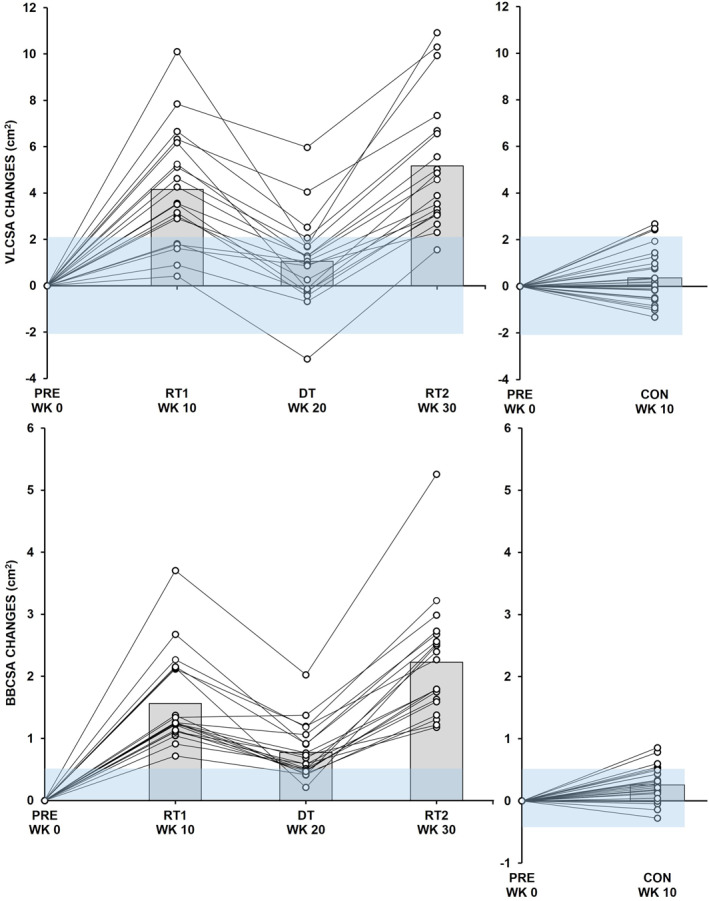
Left: Absolute changes from baseline (PRE) in vastus lateralis (VL) (above) and biceps brachii (BB) (below) muscle cross‐sectional area (CSA) during the 10‐week (WK) intervention periods of the first resistance training period (RT1), detraining period (DT), and the second resistance training period (RT2) in the experimental resistance training group. Minimal detectable change is presented as a blue area. Bars reflect the average changes for the group. Right: Absolute changes in the control group during the nontraining control period (CON).

**FIGURE 3:**
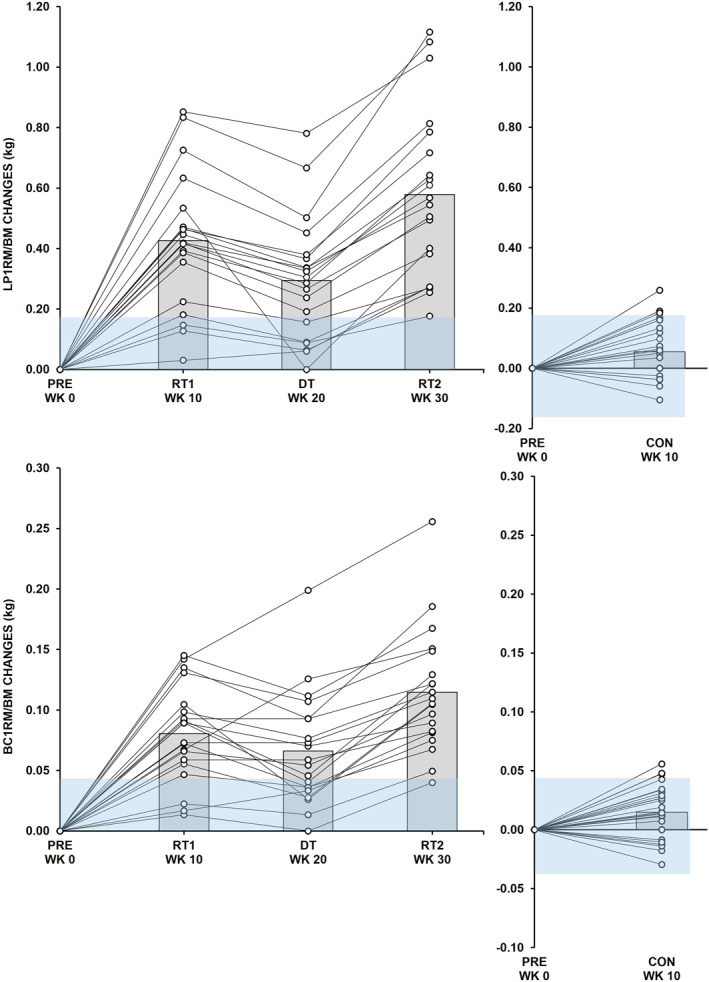
Left: Absolute changes from baseline (PRE) in leg press (LP) (above) and biceps curl (BC) (below) one repetition maximum (1RM) normalized to the body mass (BM) during the 10‐week (WK) intervention periods of the first resistance training period (RT1), detraining period (DT), and the second resistance training period (RT2) in the experimental resistance training group. Minimal detectable change is presented as a blue area. Bars reflect the average changes for the group. Right: Absolute changes in the control group during the nontraining control period (CON).

The standard deviation of individual changes within a group, that is, SD^2^ in absolute change for VLCSA, was higher in the RT group during RT1 (6.30) compared to the nontraining control group (1.19), yielding an SD_IR_ of 2.26. SD_IR_ values for other variables were as follows: BBCSA, 0.67 (RT 0.53 vs. control 0.07), LP1RM/BM, 0.21 (RT 0.05 vs. control 0.01), and BC1RM/BM, 0.0326 (RT 0.0016 vs. control 0.0005). The observed variance of individual responses (SD_IR_^2^) was 5.114 for VLCSA, with 95% confidence limits for SD_IR_^2^ ranging from 0.947 to 9.281 and a SE of 2.125. The lower confidence bounds exceeded zero, confirming meaningful individual variability beyond measurement error or chance. Furthermore, the 95% confidence limit bounds did not include zero, indicating that the difference is statistically significant. Similar results were observed in all variables as follows: for BBCSA, SD_IR_^2^ was 0.455 (95% CI: 0.117–0.793 and SE 0.172); for LP1RM/BM, SD_IR_^2^ was 0.041 (95% CI: 0.014–0.07 and SE 0.017); and for BC1RM/BM, SD_IR_^2^ was 0.001 (95% CI: < 0.001–0.002 and SE 0.001) These findings indicate that responses varied between individuals due to the RT intervention. Figures 2 and 3 present individual results alongside group‐level outcomes.

The TEΔ and MDC results were for VLCSA 0.770 and 2.135 cm^2^, for BBCSA 0.191 and 0.528 cm^2^, for LP1RM/BM (kg/kg) 0.064 and 0.176, and for BC1RM/BM 0.016 and 0.043, respectively.

### 3.2 The Correlations Between the Repeated RT Periods

**FIGURE 4:**
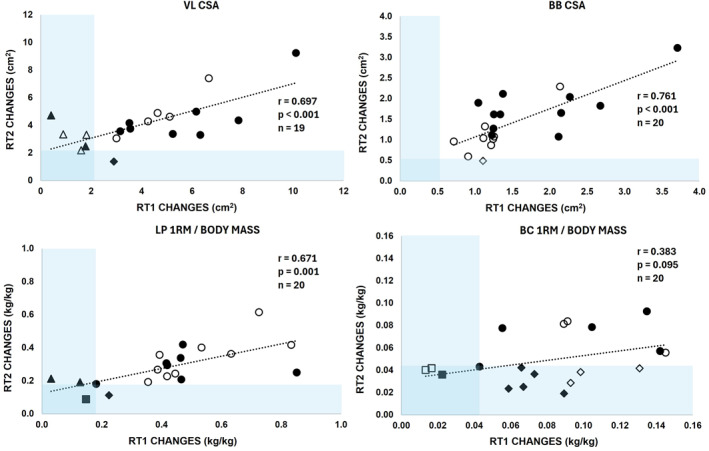
Correlations for absolute changes in vastus lateralis (VL) cross‐sectional area (CSA), biceps brachii (BB) CSA, and leg press (LP) and biceps curl (BC) one‐repetition maximum (1RM) normalized to body mass across the first (RT1, from week 0 to week 10) and the second resistance training period (RT2, from week 20 to week 30) in the experimental resistance training group. Minimal detectable change (MDC) is presented as a blue area. Symbols represent individual participant outcomes: Square = participants who did not exceed the MDC in either RT period; triangle = participants whose response did not exceed the MDC in RT1 but exceeded it in RT2; diamond = participants who exceeded the MDC during RT1 but not in RT2; and circle = participants who exceeded the MDC in both RT periods. Transparent symbols represent females, whereas solid symbols indicate males.

Statistically significant correlations (*r* = 0.671–0.761 and *p* ≤ 0.001) were observed between the two RT periods (a change from 0 to 10 weeks in RT1 and 20–30 weeks in RT2) in all variables except biceps curl 1RM/body mass (*r* = 0.383 and *p* = 0.095) (Figure 4). For VLCSA, five participants were below the MDC after the RT1, whereas one was below the MDC after the RT2. For BBCSA, none of the participants were below the MDC after RT1 and one participant fell below the MDC after RT2. For LP1RM/BM, three participants were below the MDC after the RT1 and two after the RT2, of which one was below MDC in RT1 and RT2 (a square‐shaped mark in Figure 4). In BC1RM/BM, three participants were below the MDC after RT1 and RT2, whereas eleven were below the MDC after RT2. None of the participants fell below the MDC threshold in both RT periods for more than one variable.

Five participants exhibited extreme responses in biceps muscle size, achieving changes exceeding three times the MDC in both RT periods. Similarly, two extreme responders were identified for VL muscle size and one for leg press strength. One participant demonstrated extreme responses in both leg and biceps muscle size as well as leg strength, whereas another showed extreme responses in muscle size for the legs and biceps.

### 3.3 Individuality of Detraining

**FIGURE 5:**
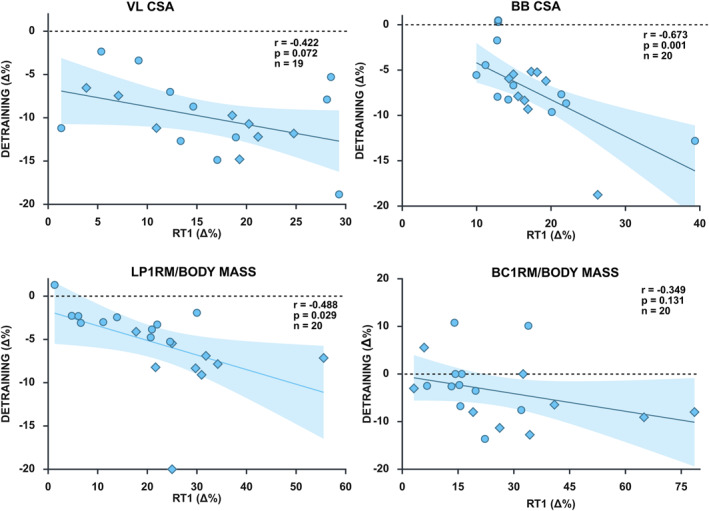
Relationships between relative changes during the first 10‐week resistance training period (RT1, from week 0 to week 10) and the 10‐week detraining period (from week 10 to week 20). Diamond symbols represent females and circle males. 1RM = one repetition maximum, BB = biceps brachii, BC = biceps curl, CSA = cross‐sectional area, LP = leg press, and VL = vastus lateralis.

Statistically significant correlations between the improvements in the first RT and the decreases in the detraining were observed for BBCSA (*r* = −0.673 and *p* = 0.001) and LP1RM/BM (*r* = −0.488 and *p* = 0.029), whereas a trend was observed in VLCSA (*r* = −0.422 and *p* = 0.072) (Figure 5). The relative decreases during detraining were more pronounced for muscle size compared to strength (VLCSA vs. LP1RM/BM: −9.94 ± 4.13% vs. −5.40 ± 4.35%, F(1,37) = 11.2, and *p* = 0.002 and BBCSA versus BC1RM/BM: −7.27 ± 3.92% versus −3.56 ± 6.76%, F(1,19) = 5.4, and *p* = 0.031).

## 4 Discussion

Muscle strength and size responses in the present repeated RT study exhibited modest to strong positive correlations, indicating that individuals with lower responses in the first RT period may also show lower responses in subsequent RT periods. Similarly, those with higher responses in the first period are more likely to have higher responsiveness during the second training period. Our results support the conclusion that individual variability in muscle size and strength responses to RT originates from heterogeneity in physiological adaptations rather than arising due to measurement error, biological variation over time, or other random noise.

To our knowledge, this is the first study to assess intraindividual variability in muscle strength and size responses during repeated RT periods. Herein, we showed positive relationships between the two identical RT interventions across all variables, except for biceps curl strength, where a statistically nonsignificant trend was observed. These positive relationships between the muscle size and strength responses of the first and second RT periods align with our hypothesis that the RT responses are reproducible. The consistent responsiveness to RT periods observed across participants, along with SD_IR_ values exceeding zero for all variables, provides strong evidence of heterogeneity in response to RT. This aligns with previous findings demonstrating substantial variability in RT‐induced changes in maximum muscle strength and muscle size among individuals (Erskine et al. 2010). Furthermore, Nunes et al. (2021) reported that skeletal muscle mass changes in an initial 12‐week RT were related to changes in a subsequent higher‐volume 12‐week RT. This consistency suggests that individual responsiveness may be influenced by genetic, molecular, or other biological factors (Roberts et al. 2018). Although identifying the specific determinants of responsiveness was beyond the scope of the present study, future research integrating physiological, molecular, and behavioral measures may help to elucidate these mechanisms.

The intra and inter individual variation in determined exercise responses could be due to the measurement error. However, the analyses of repeatability observed in the present variables suggest high precision in the estimates (Halonen et al. 2024). Therefore, the inconsistencies observed in the individual responses between the RT periods could be attributed to measurement error only to a small extent. Secondly, individual variation in responses may be due to normal physiological variation over time, independent of the effects of exercise. To account for this, the study used nontraining control group results to examine for random and biological variation. The data revealed clear muscle strength and size responses by the present RT, which exceeded, in most participants, normal fluctuations of muscle strength and size over a 10‐week follow‐up period. When considering the current finding of consistency in responses to repeated RT protocols, we can interpret these results as evidence that individual physiological characteristics largely determine RT responsiveness, rather than random factors, and that investigating the underlying physiological and environmental factors is warranted. Thus, factors, such as dietary protein intake, sleep quality, and training effort, may all contribute to the observed variation and should be systematically investigated in future studies.

From the RT group, we categorized responder subgroups based on the minimal detectable change. Nonresponders who showed a change of less than MDC over a 10‐week RT period were found for all variables. However, only one was detected after retraining for biceps brachii CSA responses. This may be because arm flexors are used less intensively in everyday life, leading to a strong muscular response through increased RT‐induced muscle activity in previously untrained participants. Some participants were identified as nonresponders in the first RT period and some in the second RT period. A total of four participants were categorized as nonresponders in both RT periods, representing approximately 20% of the present RT group. Interestingly, these nonresponders were observed only in strength variables, whereas no nonresponders were identified for muscle size across both RT periods. Importantly, none of these participants categorized as nonresponders for one variable were nonresponders for any other variable. In addition to nonresponsiveness, some very high responses were observed in the present data. Five participants showed extreme responses in both RT periods, exceeding three times MDC. One participant showed exceptional overall responsiveness, exhibiting extreme responses in all measures except for the biceps curl 1RM after the retraining period, whereas another participant exhibited extreme responses specifically in muscle size for the legs and biceps. Overall, true lower and higher responsiveness to RT is relatively rare and appears to be associated with only one trait being practiced. Ultimately, the present results show that determining muscle strength and size responsiveness based on a single RT period and a single training outcome may lead to a false interpretation about an individual's responsiveness to RT.

In this study, we showed that higher gains in muscle size and strength were followed by greater losses, suggesting that those who adapt quickly also tend to lose adaptations faster, corroborating our earlier findings (Räntilä et al. 2021). The observed associations between initial RT and detraining suggest that physiological adaptation processes to RT and detraining may occur at similar rates but in opposite directions within individuals. The consistency of responses across the present RT periods further supports this interpretation. These findings suggest stable underlying biological differences that determine individual variability in responsiveness to both RT and detraining. However, further investigation of the response heterogeneity in detraining is warranted with a repeated detraining design.

Previous studies have suggested that strength may remain elevated compared to muscle size during the detraining phase (Psilander et al. 2019; Sakugawa et al. 2019; Staron et al. 1991). Our results support these findings as muscle strength decreased statistically significantly less during detraining than muscle size. Neural adaptations, including increased motor unit firing rate and synchronization, enhanced corticospinal excitability, and improved motor control (Gabriel et al. 2006), are known to persist even when muscle mass declines, leading to better retention of muscle strength compared to hypertrophy (Andersen et al. 2005). Although neural adaptations were not determined in the present study, we can assume that they occurred during the present RT period and, thus, explain the lower detraining effects on muscle strength compared to size.

The primary objective of the present study was to investigate intraindividual variation in responses to repeated RT, which was achieved. However, the relatively small sample size to examine response heterogeneity between individuals can be acknowledged. Still, the magnitude of the RT response was similar to other studies with much larger sample sizes (Ahtiainen et al. 2016; Erskine et al. 2010). It should also be noted that the categorization of RT responsiveness is based on the distribution of the outcomes in the present group of participants. However, the results observed in the current participants can likely be replicated in another group of participants but can still only be generalized to healthy untrained younger adults. It should also be acknowledged that the present RT and detraining intervention periods were relatively short for adaptations to manifest. Studies with more extended intervention periods may be challenging to implement but are required in the future to confirm the present results. Nevertheless, systematic RT and detraining responses were observed, confirming the present conclusions. Moreover, possible determinants explaining individual response heterogeneity were not the focus of the present article but are essential to investigate in the future.

In conclusion, the current correlation results show that responsiveness to RT appears to be consistent when a similar training protocol is repeated after detraining. Thus, our results indicate that the RT‐induced muscle strength and size responses do have a physiological origin, while also being attributed to some naturally occurring measurement error, biological day‐to‐day variation, and random error. Notably, individual responses can also be noted during training breaks. Participants with greater responses to RT tended to experience a greater decline in muscle strength and size during the detraining period. This suggests that individuals who gain muscle mass to a greater extent may also lose it rapidly when training ceases. These findings justify further investigations into the physiological mechanisms and other factors underlying heterogeneity in muscle strength and size response to RT and by extending these examinations to individual consequences of training cessation.

### 4.1 Practical Implications

From a practical point of view, the following should be considered:

— It is expected that a very high heterogeneity in exercise responses can be observed, ranging from a few percent to more than 70%.
— The increase in upper and lower body muscle strength and size through resistance training exceeds measurement error or normal physiological variation over time.
— Only a small proportion of people indicated that they were nonresponsive for a particular characteristic, but no one was nonresponsive for all traits. Similarly, very few showed extreme responsiveness to resistance training.
— When resistance training was repeated after a training break, training responsiveness showed similar trends within individuals.
— Responses to detraining were also individualized, showing a higher reduction rate in those with higher responses during the previous resistance training period.

## Funding

This work was supported by the Urheiluopistosäätiö (2021 and 2022), Rehabilitation Foundation Peurunka (2021 and 2022), and Suomen Urheilututkimussäätiö (2022). J.P.A. was funded by the Research Council of Finland (2023, #357185). The funders had no role in the design of the study, in the collection, analyses, or interpretation of data, in the writing of the manuscript, or in the decision to publish the results.

## Ethics Statement

The study was approved by the University of Jyväskylä's ethics committee (857/13.00.04.00/2021) and conducted in accordance with the Declaration of Helsinki. Informed consent was obtained from all participants involved in the study.

## Conflicts of Interest

The authors declare no conflicts of interest.

## Data Availability

The raw data supporting the conclusions of this article will be made available by the corresponding author upon reasonable request.
